# Prospectively versus Retrospectively ECG-Gated 256-Slice CT Angiography to Assess Coronary Artery Bypass Grafts — Comparison of Image Quality and Radiation Dose

**DOI:** 10.1371/journal.pone.0049212

**Published:** 2012-11-07

**Authors:** Yi-Wei Lee, Ching-Ching Yang, Greta S. P. Mok, Wei-Yip Law, Cheng-Tau Su, Tung-Hsin Wu

**Affiliations:** 1 Department of Biomedical Imaging and Radiological Sciences, National Yang Ming University, Taipei, Taiwan; 2 Department of Radiology, Kaohsiung Chang Gung Memorial Hospital and Chang Gung University College of Medicine, Kaohsiung, Taiwan; 3 Department of Radiological Technology, Tzu Chi College of Technology, Hualien, Taiwan; 4 Department of Electrical and Computer Engineering, Faculty of Science and Technology, University of Macau, Macau, China; 5 Department of Radiology, Shin Kong Wu Ho-Su Memorial Hospital, Taipei, Taiwan; Virginia Tech, United States of America

## Abstract

**Objective:**

In this retrospective non-randomized cohort study, the image quality and radiation dose were compared between prospectively electrocardiogram (ECG)-gated axial (PGA) and retrospectively ECG-gated helical (RGH) techniques for the assessment of coronary artery bypass grafts using 256-slice CT.

**Methods:**

We studied 124 grafts with 577 segments in 64 patients with a heart rate (HR) <85 bpm who underwent CT coronary angiography (CTCA); 34 patients with RGH-CTCA and 30 patients with PGA-CTCA. The image quality of the bypass grafts was assessed by a 5-point scale (1 = excellent to 5 = non-diagnostic) for each segment (proximal anastomosis, proximal, middle, distal course of graft body, and distal anastomosis). Other objective image quality indices such as noise, signal-to-noise ratio (SNR) and contrast-to-noise ratios (CNR) were assessed. Radiation doses were also compared.

**Results:**

Patient characteristics of the two groups were well matched except HR. The HR of the PGA group was lower than that of the RGH group (62.0±5.0 vs. 65.7±7.4). For both groups, over 90% of segments received excellent or good image quality scores and none was non-evaluative. The image quality generally degraded as graft segment approached to distal anastomosis regardless of techniques and graft types. Image quality scores of the PGA group were better than those of the RGH group (1.51±0.53 vs. 1.73±0.62; *p*<0.001). There was no significantly difference of objective image quality between two techniques, and the effective radiation dose was significantly lower in the PGA group (7.0±1.2 mSv) than that of the RGH group (20.0±4.6 mSv) (*p*<0.001), with a 65.0% dose reduction.

**Conclusions:**

Following bypass surgery, 256-slice PGA-CTCA is superior to RGH-CTCA in limiting the radiation dose and obtaining better image quality for bypass grafts.

## Introduction

Coronary artery bypass grafting (CABG) is frequently performed to restore myocardial perfusion in patients with severe three-vessel disease or left main coronary artery (LMCA) stenosis. Although conventional coronary angiography is the current standard for the detection of graft patency and stenosis, it is associated with a small but non-negligible risk of complications such as stroke, myocardial infarction, pericardial tamponade, patient discomfort, and costs of hospital stay. With advances in the multi-detector computed tomography (MDCT), the great improvement in spatial and temporal image resolution has allowed non-invasive evaluation of cardiac anatomy, coronary arteries, stents and bypass grafts [1], [2], [3], [4]. Computed tomography coronary angiography (CTCA) using 64-slice CT is highly accurate in the detection of graft occlusion as well as stenosis, but still associates with the cost of high radiation and contrast doses [2], [3], [4], [5], [6]. Based on the recent guideline, CTCA using 64-slice CT is considered “appropriate” for symptomatic patients with prior coronary bypass surgery [2]. Thus, CTCA assessment of CABG and anastomosis following surgery is expected to be a noninvasive alternative, and may demonstrate clinical benefit in selected patients. However, radiation exposure with CTCA has become an important issue due to the increased risk of cancer induction [7], [8], [9], [10]. In an average of all age groups, an estimated additional lifetime risk for developing cancer after 10 mSv exposure is approximately one in 2000 [11]. Because the wider scan coverage required to access the entire heart and graft course for CABG follow-up, MDCT exposes patients to a much higher radiation dose, approximately twice than that of cardiac CT angiography (9–19 mSv) [2], [12], [13].

Recently, prospectively electrocardiogram (ECG)-gated axial (PGA) CTCA, especially with body mass index (BMI) adapted protocol, has allowed marked reduction of the radiation dose to <6 mSv, resulting in a substantial reduction in dose of 52% to 85% [8], [9], [14] as compared to retrospectively ECG-gated helical (RGH) CTCA. Image quality of coronary artery with PGA is reported to be either better [8] or equivalent to [10], [15] that with RGH technique in 64-slice CT. However, studies suggest that PGA-CTCA using the current 64-slice CT remains limited by heart rate (HR) and heart rate variability (HRV) [15]. The recent introduction of the 256-slice CT scanner (Brilliance iCT; Philips Medical Systems, Eindhoven, Netherlands) with an 80.0 mm detector array has allowed a larger z-axial coverage. A thorough assessment on the effects of wide-coveraged 256-slice CT for patients with CABG has not been well addressed. The purpose of this study was to compare the image quality, radiation dose, and graft vessel accessibility for patients with prior coronary bypass surgery using a 256-slice CT scanner with both PGA and RGH techniques.

## Materials and Methods

### Study Population

The study protocol was approved by the Institutional Review Board of Shin Kong Wu Ho-Su Memorial Hospital, Taipei, Taiwan, and the need for informed consent was waived because it was a retrospective study and the data were analyzed anonymously. We reviewed the hospital database for patients with prior coronary bypass surgery and received CTCA from January 2010 to June 2012. Totally we selected 65 consecutive patients: 34 patients with RGH-CTCA and 30 patients with PGA-CTCA. Patients were excluded from the exam if they reported the following conditions: (*i*) allergy to iodinated contrast agent, (*ii*) renal insufficiency (blood creatinine level >1.27 mg/dL or glomerular filtration rate <60 mL/min/1.73 m^2^), (*iii*) non-sinus rhythm, (*iv*) history of asthma or chronic obstructive pulmonary disease, (*v*) implanted pacemaker or automatic implantable cardioverter defibrillator, (*vi*) hemodynamic instability, or (*vii*) pregnancy. Most CTCA scans were performed for clinical suspicion of vascular insufficiency of coronary bypass grafts or native coronary arteries. One patient who received an RGH-CTCA scan was excluded from the present study because all bypass grafts were occluded.

### 256-slice CTCA Examination

All CTCA examinations were performed using the Brilliance iCT 256-slice CT scanner (Philips Medical Systems, Eindhoven, Netherlands). Iterative reconstruction algorithm was not available in this model and all images were reconstructed using filtered back projection method. In addition, there is no 100 kVp option available in this scanner. Scanning was conducted in a craniocaudal direction covering a region from the level of the aortic arch to the diaphragm in the case of venous grafts and from the subclavian artery in the case of in-situ internal thoracic arterial grafts. For both RGH and PGA studies, the scanning delay was determined using an automatic bolus tracking technique. A single unenhanced scan was obtained at the level of the aortic root. This scan was used to place a circular region-of-interest (ROI) with 10 mm diameter inside the lumen of the ascending aorta. Then, based on the weight of the patients, 70–90 mL of a nonionic contrast medium (Optiray 350, Tyco Healthcare, Montreal, Quebec, Canada) was injected at a flow rate of 5 mL/s, followed by a 30 mL bolus of saline at the same rate using a dual-head injector (Stellant D; Medrad, Warrendale, PA, USA). As soon as the intensity in the ROI exceeded 90 Hounsfield units (HU), scanning was initiated. The 256-slice scanner incorporates real-time arrhythmia management capability that enables the X-ray acquisition to be paused upon the detection of ectopy during a PGA scan and then to be resumed at the same axial location once normal sinus rhythm has returned.

Images were reconstructed with a slice thickness of 0.9 mm [14], [16], [17], a reconstruction increment of 0.45 mm, and a medium soft-tissue convolution kernel (XCB). The reconstructed matrix size was 512×512. The field-of-view was manually adjusted to encompass the whole heart and bypass grafts. All images were transferred to a separate workstation equipped with the post-processing software (Extended Brilliance Workspace 4.0; Philips).

To increase patient throughput and based on our previous reports [14], beta-blockers were not administered unless the patient’s HR was >80 bpm (beats per minute) prior to CTCA. Those patients were administered 20 mg of propranolol (Inderal, AstraZeneca UK Limited, Cheshire, UK) every 45–60 minutes orally if they have no documented contraindications, and then returned for CTCA after their HR decreased to <80 bpm. All patients received a single dose of 0.6 mg sublingual nitroglycerin (Nitrostat, Pfizer Pharma, Vega Baja, Puerto Rico) 2 minutes prior to the acquisition of non-contrast localization images. The assignment of the patients to the imaging protocol was not randomized, but based on the patients’ HR. For all patients with a prescan HR ≤70 bpm, PGA acquisitions with X-ray trigger centered at 75%±5% phase tolerance of the R-R interval, i.e., cardiac cycle, were used. Patients whose prescan HR >70 bpm were imaged with RGH acquisitions.

The acquisition parameters for both PGA and RGH acquisition protocols are summarized in Table 1. For PGA acquisition, 3 types of detector collimation, i.e., 96×0.625 mm, 112×0.625 mm, 128×0.625 mm, were adapted by the scanner automatically. The other scanning parameters were as followed: gantry rotation time, 270 ms; pitch, 1. The tube voltage was 120 kV and an effective tube current-time product of 150 to 300 mAs was applied according to the patient’s body weight. For RGH acquisition, the following parameters were used: 128×0.625 mm detector collimation, 256×0.625 mm slice collimation by means of a dynamic z-focal spot for double sampling, and 270 ms gantry rotation time. Applying the common half scan reconstruction techniques allowed a minimum temporal resolution of up to 135 ms. HR-dependent pitch was set at 0.16 for patients with HR ≤62 bpm and 0.18 for patients with HR >62 bpm. The tube voltage was 120 kV and an effective tube current-time product of 600 to 1300 mAs was applied according to the patient’s body weight. To produce the best possible image quality and to assess the systolic ventricular function and perfusion with the RGH technique, ECG-based tube current modulation was turned off for all patients.

**Table 1:** 256-slice CT Scan Parameters, Estimated Patient Radiation Dose, and Objective Image Quality Parameters.

	RGH (*n* = 34)	PGA (*n* = 30)	*p* value
Tube voltage (kV)	120	120	N/A
Tube current (mA)	639.9±113.4	779.6±149.7	<0.001*
ffective mAs	1036.4±199.0	210.5±40.4	<0.001*
can time (s)	6.6±0.8	7.3±1.2	0.007*
TDI_vol_ (mGy)	69.6±14.3	24.0±3.6	<0.001*
can length (cm)	206.4±36.8	208.5±22.8	0.781
LP (mGy ⋅ cm)	1427.1±325.3	500.5±88.6	<0.001*
ffective dose (mSv)	20.0±4.6	7.0±1.2	<0.001*
Image Quality Parameter			
mage noise (HU)	33.36±5.26	34.59±6.83	0.418
ignal-to-noise ratio (SNR)	12.52±2.65	12.70±2.58	0.784
ontrast- to-noise ratio (CNR)	9.12±2.29	9.10±1.99	0.969

CABG, coronary artery bypass grafting; PGA, prospectively ECG-gated axial; RGH, retrospectively ECG-gated helical; CTDI_vol_, the volume CT dose index; DLP, dose-length product.

Data are presented as mean ± standard deviation for continuous variables and number (percentage) for categorical variables.

Mann-Whitney U test was used.

* : *p*<0.05.

### CT Data Post-processing and Analysis

Independent experienced radiology technologists and one cardiovascular radiologist selected the best cardiac phase to create volume rendering, curved multiplanar reformation, and appropriate subvolume oblique images that included the heart and course of bypass grafts for measurements of image quality parameters. For RGH acquisition, 20 datasets were first reconstructed in 5% steps from 0% to 95% of the R-R interval using a 512×512 matrix. After the technologists and the radiologist determined the preliminary optimal R-R intervals, further reconstructions were performed with 1% step in those selected R-R intervals to locate finer optimal intervals. For PGA acquisition, optimal R-R intervals were determined by the same experts from 11 datasets that were reconstructed in 1% steps from 70% to 80% of the R-R interval. Five segments (proximal anastomosis, proximal, middle, distal course of graft body, and distal anastomosis) were classified for free arterial or venous grafts and 4 segments (proximal, middle, distal course of graft body, and distal anastomosis) were classified for in-situ arterial grafts. The opacified graft body was further divided into three parts with equal length on curved-multiplanar-reformatted images. “Distal course” meant that the graft segment was closer to the heart and vice versa.

Measurements of the image quality of each segment for the non-occluded bypass grafts were accomplished in random order by two independent cardiovascular radiologists, each with >5 years experience, who knew the numbers, types, and sites of the anastomoses of the grafts but were blinded to other patient and CT scan information. Graft occlusion was excluded from image quality evaluation and was defined as absence of contrast medium along the course of the graft or through the anastomosis to the native distal artery. There was no sequential or T-graft included in this retrospective study. To estimate HRV, the length of each heart beat during data acquisition was measured for each patient and HRV was then calculated as the standard deviation from the mean HR.

**Figure 1:**
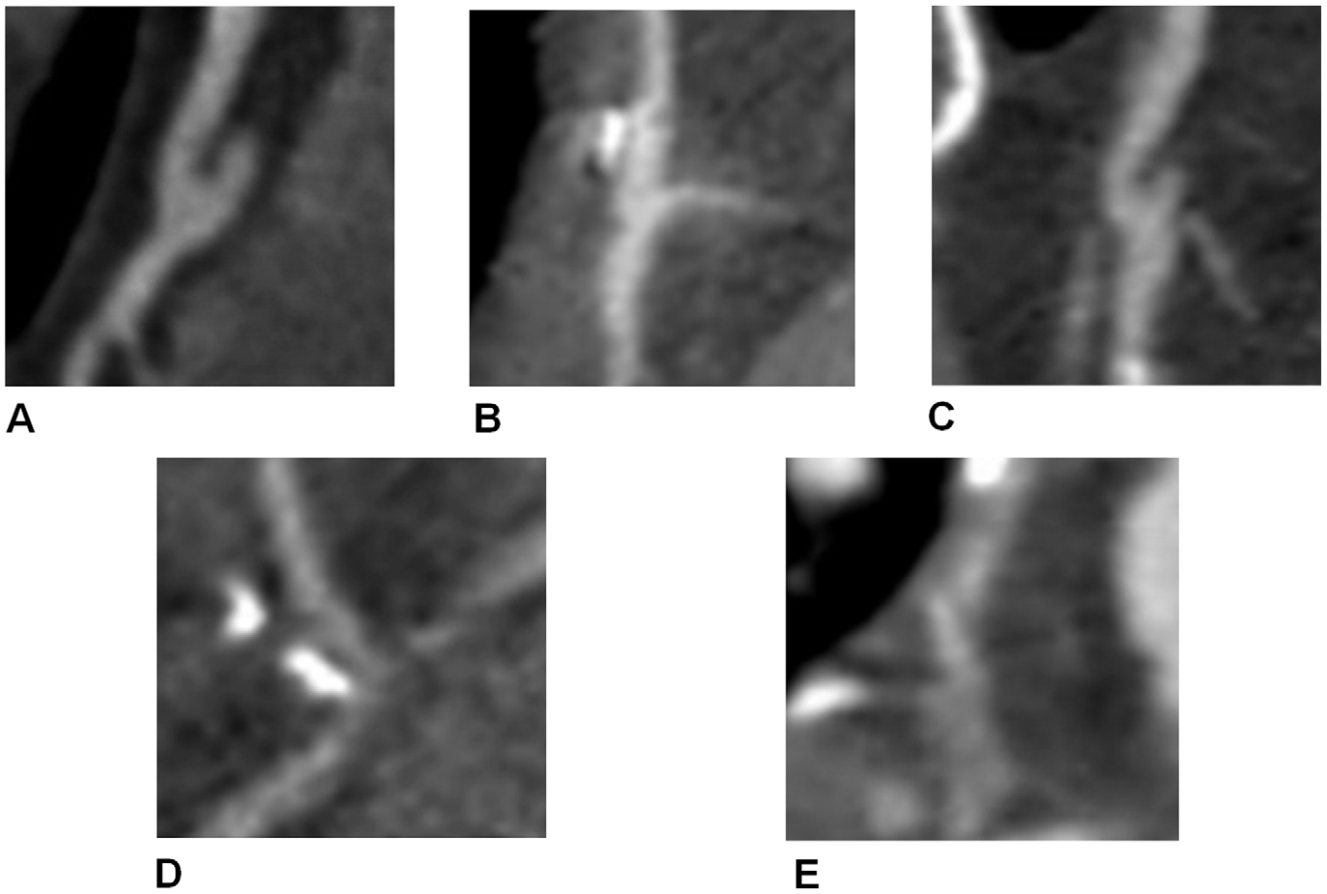
Curved multi-planar reconstruction images of the anastomotic sites in the distal arterial bypass graft illustrated the use of five-point image quality score. (A) Score 1: No motion artifacts or noise-related blurring, and no structural discontinuity of the segment. (B) Score 2: Minor motion artifacts, and no structural discontinuity of the segment. (C) Score 3: Moderate motion artifacts and moderate blurring without structure discontinuity of the segment. (D) Score 4: Severe motion artifacts with mild structural discontinuity. (E) Score 5: Severe artifacts with discontinuity or doubling in the course of the segment.

We performed semi-quantitative analysis by using a 5-point ranking scale as follows: a score of 1 = no motion artifacts or noise-related blurring, and no structural discontinuity of the segment; a score of 2 = minor motion artifacts, and no structural discontinuity of the segment; a score of 3 = moderate motion artifacts and moderate blurring without structure discontinuity of the segment; a score of 4 = severe motion artifacts with mild structural discontinuity; a score of 5 = severe artifacts with discontinuity or doubling in the course of the segment (Figure 1). A score of 3 or lower was considered acceptable for routine clinical diagnostic purposes. The subjective assessment of native coronary artery was not performed because of diffuse, calcified, and atherosclerotic disease in our study. Besides the subject image quality evaluation, the mean CT-attenuation values in HU, noise, *i.e.*, standard deviation (SD) of the mean CT-attenuation values, contrast-to-noise ratios (CNR) and signal-to-noise ratios (SNR) were also measured by putting a circular ROI of 100 mm^2^ at the ascending aorta (AAo) at the level of the LMCA and a circular ROI of 20 mm^2^ at the interventricular septum (IVS) at the level of mid-portion of LV on axial reconstructed images. The CNR and SNR were defined as follows:
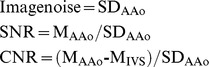



### Radiation Dose

Radiation dose estimates were determined using the volume CT dose index (CTDI_vol_) in Gy, as provided on the scanner console, and effective dose was expressed in mSv. The dose-length product (DLP) was defined as the volume CT dose index multiplied by scan length, and was an indicator of the integrated radiation dose of an entire CT examination. Calculation of estimated effective dose was obtained by multiplying the DLP by a conversion factor, k (0.014 mSv ⋅ mGy^−1^ ⋅ cm^−1^) as recommended by the American Association of Physicists in Medicine (AAPM) Report 96 [18].

### Statistical Analysis

Data were presented as mean ± standard deviation (SD) for continuous variables and number (percentage) for categorical variables. Comparisons between the PGA and RGH techniques were performed by Mann-Whitney U test for continuous covariates and by χ^2^ test for categorical variables. Cohen's kappa coefficient was calculated to examine the inter-observer agreement for image quality assessment. Five levels of kappa values are defined as follows: very poor reliability (kappa value ≤0.20); poor reliability (0.21 to 0.40); fair reliability (0.41 to 0.60); good reliability (0.61 to 0.80); excellent reliability (>0.80).

Wilcoxon signed rank test was used to evaluate the difference in the image quality between PGA and RGH techniques for all segments together and separately for each vessel. Pearson’s correlation analysis was performed to estimate strength of correlations between motion scores of all segments and mean HR. Five levels of Pearson’s correlation coefficient (r) are defined as: very weak (r≤0.2); weak (0.2<r≤0.4); moderate (0.4<r≤0.6), strong (0.6<r≤0.8), and very strong (r>0.8). All statistical analyses were performed with NCSS statistical software, version 2007 (NCSS; Kaysville, UT, USA). A 2-tailed *p* value of <0.05 was considered statistically significant.

## Results

The CT scan parameters and patient characteristics of the 64 patients are summarized in Table 1 and Table 2. The two groups showed no significant differences with respect to age, sex, BMI, HRV and time interval between CABG surgery and CTCA (*p*>0.05 for all). The HR of the PGA group (62.0±5.0 bpm) was significantly lower than that of the RGH group (65.7±7.4 bpm). The tube voltage and z-axis scan coverage were not significantly different between the two groups. However, tube current was significantly higher in the PGA group (779.6±149.7 mA) than in the RGH group (639.9±113.4 mA) by 21.8% (*p*<0.001).

**Table 2:** Patient Characteristics.

	RGH (*n* = 34)	PGA (*n* = 30)	*p* value
Age (years)	68.4±10.8	69.3±9.4	0.712
Male gender	28 (82.4%)	27 (90.0%)	0.380+
Body mass index (BMI) (kg/m^2^)	26.4±3.6	25.8±2.2	0.456
Mean heart rate (HR) (bpm)	65.7±7.4(median 66, interquartile range 11)	62.0±5.0(median 63, interquartile range 6)	0.023*
Heart rate variability (HRV) (bpm)	1.4±1.0	1.0±0.5	0.055
Received beta-blockers	2 (5.9%)	2 (6.7%)	0.897+
Time interval after CABG surgery (months)	60.4±23.4	58.1±31.9	0.760

CABG, coronary artery bypass grafting; PGA, prospectively ECG-gated axial; RGH, retrospectively ECG-gated helical.

Data are presented as mean ± standard deviation for continuous variables and number (percentage) for categorical variables.

+ = Chi-square test.

* : *p*<0.05.

We excluded 27 occluded grafts from the image quality assessment. Thus, we evaluated 62 grafts in the PGA group (21 left internal mammary artery (LIMA) and 41 saphenous vein (SV) grafts) and 62 grafts in RGH group (22 LIMA and 40 SV grafts). No segment was further excluded in these 124 grafts. We then assessed the image quality for 289 and 288 segments in PGA and RGH groups respectively.

### Comparison of Objective Image Quality

The quantitative image quality parameters of this CTCA study are summarized in Table 1. There were no significant differences in CNR, SNR and image noise levels between the two groups.

### Comparison of Subjective Image Quality

**Figure 2:**
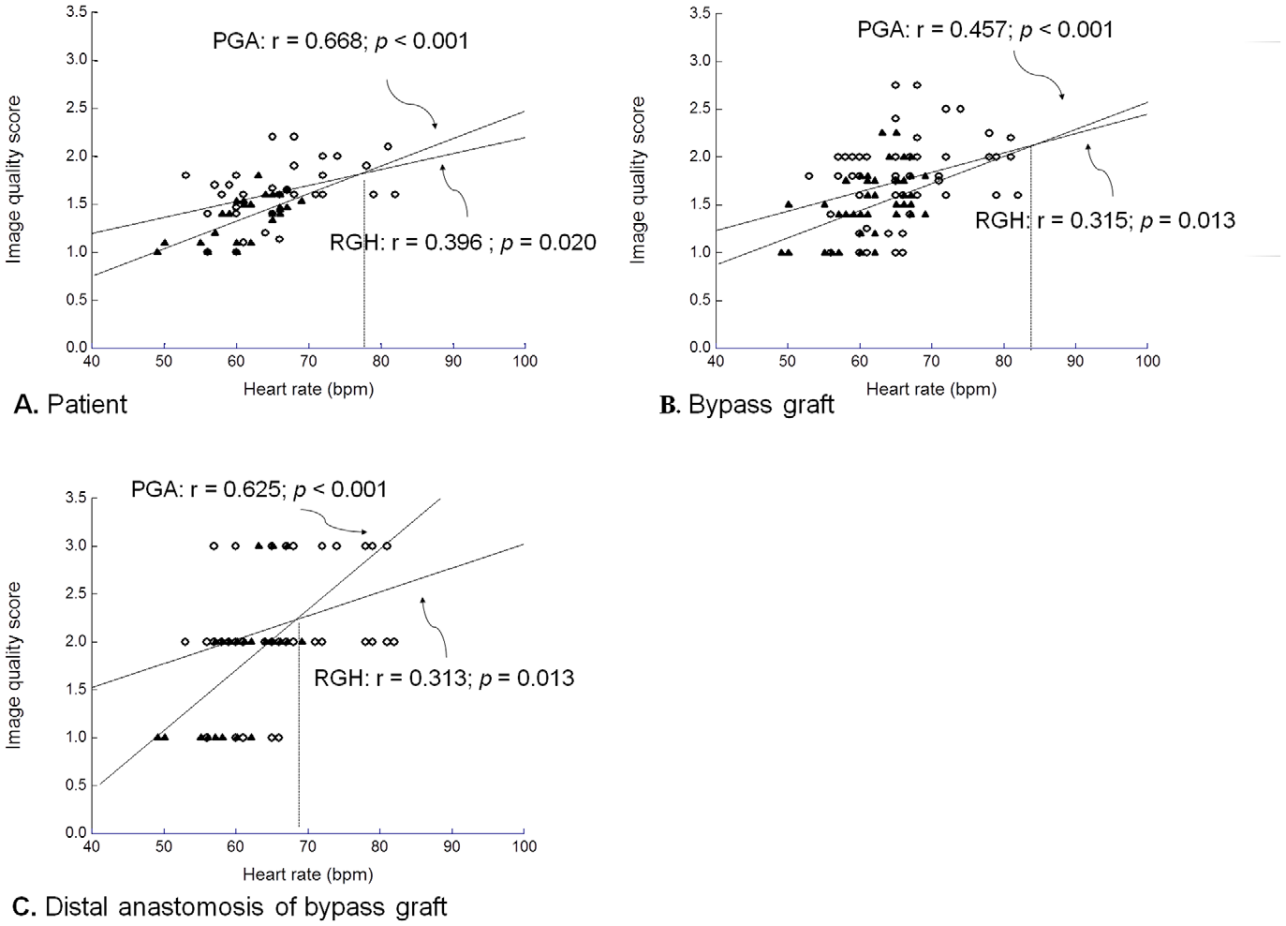
Scatter plot of heart rate (HR) in RGH group and PGA group as against the image quality scores for (A) patient-based, (B) bypass graft-based and (C) distal anastomosis of bypass graft-based analysis (Results of other segments were similar and were not shown here). Hollow circles indicate data for RGH, solid triangles indicate data for PGA, and lines represent linear regression model.

All 64 patients were in sinus rhythm and a total of 577 graft segments were evaluated. The overall inter-observer agreement for image quality scoring was excellent (κ = 0.87). The correlations between image quality score and HR are illustrated in Figure 2. There was significantly higher correlation between image quality score and HR in the PGA group regardless if the analysis was based on patients, grafts or segments of grafts. The correlation value was highest for patient-based comparison (r = 0.668; *p*<0.001) in PGA group.

**Figure 3:**
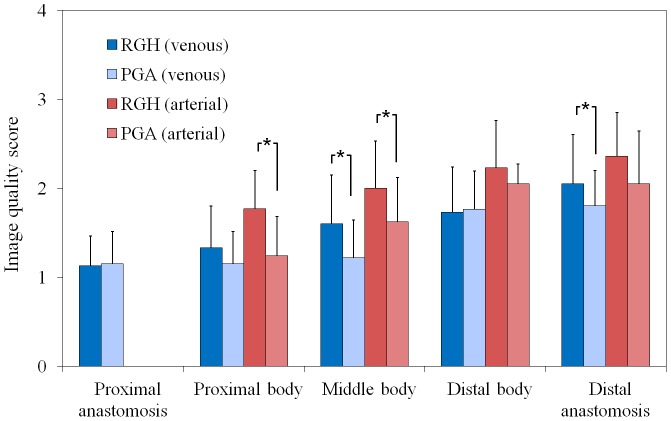
Bar chart demonstrated mean image quality scores of arterial and venous grafts for each segment in RGH and PGA techniques. (*: *p*<0.05).

The image quality scores of the two techniques are presented in Table 3 and Figure 3. Overall, 50.9% and 36.5% of segments received a score of 1, i.e., best quality image for PGA and RGH techniques, respectively. Notably, none of the CABG segments was non-diagnostic (score = 4 or 5) in both groups. The PGA group had better subjective image quality of all segments for arterial and venous bypass grafts. There was significant difference of image quality in middle body and distal anastomosis of SV grafts, proximal body and middle body of LIMA grafts in segment-to-segment comparison between two groups (Table 4). There was no inconsistent contrast agent filling of bypass grafts in the two groups. The image quality generally degraded as the segment approached to the distal anastomosis in arterial and venous bypass grafts for both groups.

**Table 3:** Subjective Image Quality Scores for All Segments for RGH and PGA Protocols.

		RGH						PGA				
Graft type/Graft segment		Score1 (%)	Score2 (%)	Score3 (%)	Score4 (%)		Score5 (%)	Score1 (%)	Score2 (%)	Score3 (%)	Score4 (%)	Score5 (%)
**Venous graft**												
Proximal anastomosis	87.5 (35/40)		12.5 (5/40)	0.0 (0/40)	0.0 (0/40)	0.0 (0/40)		85.4 (35/41)	14.6 (6/41)	0.0 (0/41)	0.0 (0/41)	0.0 (0/41)
Proximal body	67.5 (27/40)		32.5 (13/40)	0.0 (0/40)	0.0 (0/40)	0.0 (0/40)		85.4 (35/41)	14.6 (6/41)	0.0 (0/41)	0.0 (0/41)	0.0 (0/41)
Middle body	42.5 (17/40)		55.0 (22/40)	2.5 (1/40)	0.0 (0/40)	0.0 (0/40)		78.0 (32/41)	22.0 (9/41)	0.0 (0/41)	0.0 (0/41)	0.0 (0/41)
Distal body	30.0 (12/40)		67.5 (27/40)	2.5 (1/40)	0.0 (0/40)	0.0 (0/40)		24.4 (10/41)	75.6 (31/41)	0.0 (0/41)	0.0 (0/41)	0.0 (0/41)
Distal anastomosis	12.5 (5/40)		70.0 (28/40)	17.5 (7/40)	0.0 (0/40)	0.0 (0/40)		19.5 (8/41)	80.5 (33/41)	0.0 (0/41)	0.0 (0/41)	0.0 (0/41)
**Arterial graft**												
Proximal body	22.7 (5/22)		77.3 (17/22)	0.0 (0/22)	0.0 (0/22)	0.0 (0/22)		76.2 (16/21)	23.8 (5/21)	0.0 (0/21)	0.0 (0/21)	0.0 (0/21)
Middle body	13.6 (3/22)		72.7 (16/22)	13.6 (3/22)	0.0 (0/22)	0.0 (0/22)		38.1 (8/21)	61.9 (13/21)	0.0 (0/21)	0.0 (0/21)	0.0 (0/21)
Distal body	4.5 (1/22)		68.2 (15/22)	27.3 (6/22)	0.0 (0/22)	0.0 (0/22)		0.0 (0/21)	95.2 (20/21)	4.8 (1/21)	0.0 (0/21)	0.0 (0/21)
Distal anastomosis	0.0 (0/22)		63.6 (14/22)	36.4 (8/22)	0.0 (0/22)	0.0 (0/22)		14.3 (3/21)	66.7 (14/21)	19.0 (4/21)	0.0 (0/21)	0.0 (0/21)
**All graft segments**	36.5 (105/288)		54.5 (157/288)	9.0 (26/288)	0.0 (0/288)	0.0 (0/288)		50.9 (147/289)	47.4 (137/289)	1.7 (5/289)	0.0 (0/289)	0.0 (0/289)

PGA, prospectively ECG-gated axial; RGH, retrospectively ECG-gated helical.

Data in parentheses are raw data used to calculate the percentages. Image quality scores are obtained at optimal R-R reconstruction interval.

**Table 4:** Comparison of Subjective Image Quality Scores for All Segments for RGH and PGA Protocols.

Graft type/Graft segment	RGH	PGA	*p* value
**Venous graft**	1.56±0.58	1.41±0.49	0.013*
roximal anastomosis	1.13±0.33	1.15±0.36	0.787
roximal body	1.33±0.47	1.15±0.36	0.060
iddle body	1.60±0.55	1.22±0.42	0.001*
istal body	1.73±0.51	1.76±0.43	0.717
istal anastomosis	2.05±0.55	1.80±0.40	0.031*
**Arterial graft**	2.09±0.54	1.74±0.56	<0.001*
roximal body	1.77±0.43	1.24±0.44	<0.001*
iddle body	2.00±0.53	1.62±0.50	0.026*
istal body	2.23±0.53	2.05±0.22	0.136
istal anastomosis	2.36±0.49	2.05±0.59	0.079
**All graft segments**	1.73±0.62	1.51±0.53	<0.001*

* : *p*<0.05.

### Comparison of Effective Radiation Dose

**Figure 4:**
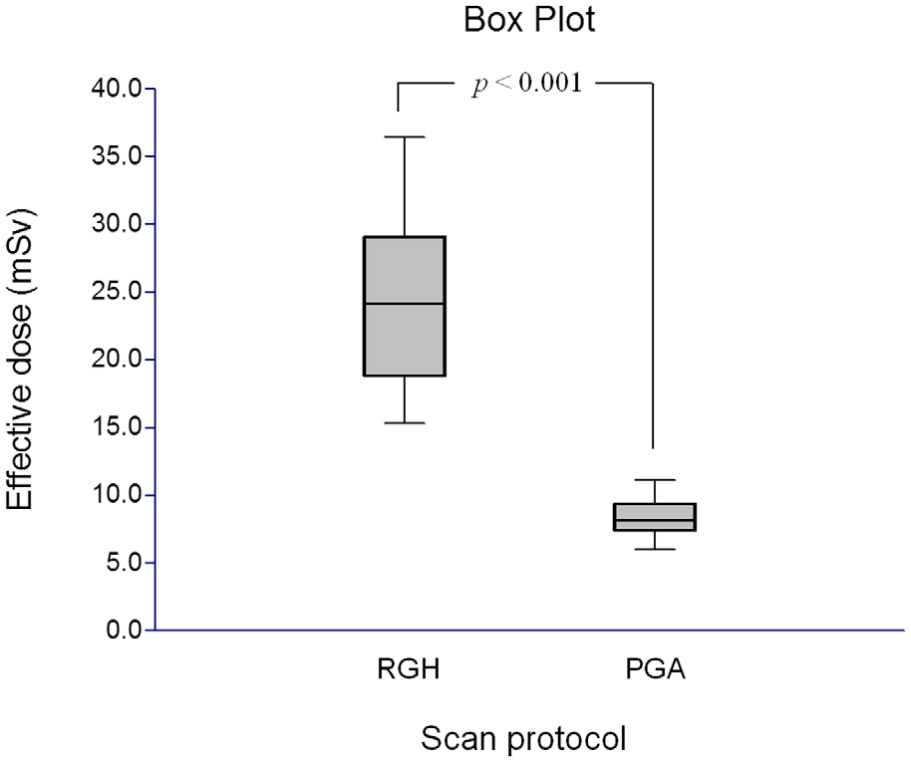
Box-and-Whisker plot showed the effective radiation doses of PGA group vs. RGH group. Box  = 1st to 3rd quartiles, mid-line  =  median and whiskers  =  minimum and maximum values.

Effective mAs, CTDI_vol_, DLP and effective dose in the RGH group were significantly greater than those in the PGA group (*p*<0.001 for all). The effective radiation doses were 7.0±1.2 mSv and 20.0±4.6 mSv for PGA group and RGH group respectively (Figure 4). Compared with the RGH group, the PGA group had an average of 65.0% reduction in the radiation dose.

## Discussion

With technical advances provided by the recently introduced 256-slice CT scanner, such as faster gantry rotation speed, larger detector coverage, and increased X-ray tube power, this device is potentially able to image patients with higher HR, higher HRV and provide wider scan length [14]. As the scan times with the 256-slice CT are substantially shorter, it reduces the susceptibility to the effects of ectopy, HRV and respiratory intolerance. However, the potential advantages of CTCA following CABG have to be weighed against the potential hazards associated with increasing ionizing radiation [7], [8], [9], [10]. Einstein *et al.* reported that the lifetime cancer risk estimates for standard RGH-CTCA varied from 1 in 143 for a 20-year-old woman to 1 in 3261 for an 80-year-old man and with the use of ECG-gated controlled tube current modulation for reducing radiation dose, the risk estimates decreased to 1 in 219 and 1 in 5017, respectively [7]. Because wider scan coverage is required to access the entire heart and graft course of CTCA following CABG, the radiation dose is approximately double as compared to the standard cardiac CT angiography [12], [13]. Therefore, CTCA examination parameters should be considered and set in accordance with the as-low-as-reasonably-achievable (ALARA) strategy without compromise of diagnostic image quality.

In this study, we found that the percentages of good and excellent image quality were 98.3% and 91% in PGA and RGH groups respectively, better than those in the documented results (80–90%) using 64-slice CT [1], [13]. The bypass grafts were fully assessable and no respiratory or motion artifacts were detected regardless of techniques. In addition, no stair-step artifact or inconsistent contrast agent filling of bypass grafts was noted in PGA group. While providing better image quality of arterial and venous bypass grafts than RGH, PGA was associated with a 65.0% reduction in effective radiation dose. Thus, we can take advantage of the significant dose reduction of the PGA technique for 256-slice CT in patients with steady and lower HR.

Previously a study showed that for a 16-slice CT, a substantial number of scanned grafts failed to provide diagnostic values (up to 22%) for patients with HR <70 bpm [19]. Justifications for non-assessability included cardiac motion, respiratory artifacts, poor opacification, prominent image noise and the presence of surgical clips. However, increased spatial and temporal resolution, which reduces acquisition time, has improved the rate of fully assessable scanned grafts, including distal anastomoses using 64-slice CT; 87–100% of grafts have been reported assessable using 64-slice CT and 78–100% for 16-detector CT for patients with HR<62–72 [5]. However, with the 256-slice CT in this study, no CABG segment was non-evaluative using both PGA and RGH techniques as compared to 1.5% as reported by Machida H *et al*. for a 64-slice CT with PGA technique [13], indicating that PGA may be particularly favorable for 256-slice CT due to its higher temporal resolution and wider z-axis coverage as compared to 64-slice CT.

Compared to previous studies with reported mean scan durations of 12.5–20 s for 16- and 64-slice CT [5], [6], scan times with the 256-slice CT were 50% to 70% shorter, i.e., 6.6–7.3 s. Less significant, yet still important, scan duration was reduced approximately 50% as compared to 40.0 mm z-coverage of 64-slice CT. While still requiring more than one axial step with axial coverage greater than 80 mm, the resulting transition zone and possibility for stair-step artifact has not been associated with non-diagnostic images as noted in previous studies with 40.0 mm of detector coverage [6], [13], [15]. Unsynchronized movement of proximal and distal anastomosis (usually for RCA and/or LCx) in vein grafts, as well as the blood flow velocity and vessel displacement during cardiac twisting contraction, may cause the image quality of the middle course of graft body and distal anastomosis more prone to be deteriorated. The PGA technique in 256-slice CT with large z-axis coverage can reduce incidence of ectopy, respiratory intolerance and motion artifacts owing to the continuous table movement in RGH acquisition. Besides, BMI is also an important factor influencing the image quality of CT studies. A high BMI unfavorably affects image quality with poorer vessel opacification [20]. Higher tube current used and relatively lower BMI in the PGA group might compensate for the higher image noise in thicker body parts. These factors may explain why PGA group has better image quality at middle body and distal anastomosis of venous grafts, proximal body and middle body of LIMA grafts as compared to that of the RGH group. However, it is also possible that the difference is because the mean HR is lower for PGA group than that of RGH group, as indicated in this study that image quality was inversely correlated with HR (Figure 2).

A significant inverse correlation between mean HR and image quality is observed in our study. The result is similar to the reported studies on native coronary artery with 16-, 64- and 256-slice CT [14], [21], [22], [23]. Furthermore, the image quality of bypass grafts became worse as the segment became closer to distal anastomosis regardless of techniques and graft types. Therefore, cardiac twisting contraction still influences the image quality and limits the assessability of distal anastomosis, especially for arterial grafts. To the best of our knowledge, this is the first report on the relationship between mean HR, bypass segment and image quality for CTCA following bypass graft surgery using 256-slice CT (Figure 2). The correlation value was highest for HR vs. patient-basis (r = 0.668; *p*<0.001) in the PGA group. Although there was a mean HR difference of 4 bpm between the RGH (65.7±7.4) and the PGA (62.0±5.0) groups, which might favor the PGA results. However, as indicated from Figure 2, the PGA group might still have better image quality even with similar mean HR (66 bpm) as in the RGH group). In addition, lower HRV was described as strongly associated with optimal image quality from other studies [14], [22]. Although the correlation between image quality score and HRV was not assessed due to the small population of the recruited subjects and low HRV of our study, the sufficiently low HRV in both groups, specifically 1.0±0.5 bpm for the PGA group and 1.4±1.0 bpm for the RGH group, may explain our encouraging results regarding motion and stair-step artifacts. Our study indicates that PGA technique should be recommended for patients with scan HR <68 bpm and low HRV to lower patient radiation dose and provide better image quality of bypass grafts. In a recent 256-slice CTCA study on native coronary artery [23], Hou et al. also suggested that PGA-CTCA could maintain image quality comparable to the RGH technique with 73% dose reduction for patients with HR up to 75 bpm and low HRV. Thus, 256-slice PGA-CTCA should be superior to RGH-CTCA in further limiting the radiation dose and obtaining better non-invasive comprehensive investigation of bypass grafts, recipient vessels and non-grafted vessels for CABG patients with HR <68 and low HRV.

Also, to decrease the amount of radiation, the 256-slice CT can automatically choose an optimal detector collimation to minimize the number of steps and reduce the amount of z-overscan. The real-time arrhythmia management capability further reduces radiation dose as exposure only occurs during normal sinus rhythm. In our analysis, the PGA technique was associated with a 65.0% reduction in effective dose as compared to RGH (PGA, 7.0 mSv; RGH, 20.0 mSv), while maintaining a similar (image noise, SNR, CNR) or even better image quality (image quality score). This finding is in agreement with prior reports using 64-slice CT [8], [10]. Earls *et al.* reported a much lower mean effective dose for CTCA after bypass surgery using 64-slice CT with PGA (6.4±2.3 mSv) than with RGH (30.8±4.8 mSv). However, that study included 100 kV protocol and the percentage of diagnostic segments was not reported. Moreover, more contrast medium (80–100 ml) and β-blocker were use (73.1%, 102.1±52.4 mg metoprolol) as compared with our study [24]. The dose associated with PGA was even lower than that of the RGH technique with an optimal ECG pulsing window for low HR patients, i.e., 18.2 mSv, as reported by Shuman WP *et al*. [10]. Thus, the radiation dose with PGA is comparable to, or even lower than doses associated with conventional coronary angiography for following CABG, which range from 8 to 15 mSv [2], [25].

Low tube voltage of 100 kV was shown to reduce the radiation dose exposure without deterioration in the image quality and diagnostic accuracy of CTCA for native coronary arteries [26], [27]. Combining the 100 kV protocol with prospective ECG-gating would be the most effective approach to minimize radiation exposure (>86% reduction) if coronary arteries evaluation is the only interest [28]. Abada *et al.* reported that the combined effects of low tube voltage (80 kV) and ECG-gated tube current modulation can reduce radiation exposure up to 88% in slim patients without impairing image quality, but the 80 kV setting is probably suboptimal for patients with a normal BMI [29]. Moreover, for obese patients with a high BMI, the 100 kV voltage setting results in a low CNR, and high image noise levels [30]. There are few reports on 100 kV CTCA for CABG follow-up [24]. While 100 kV option is not available for our scanner, further study to evaluate the diagnostic accuracy and image quality of 100 kV PGA/RGH-CTCA using a newer model of 256-slice CT is necessitated.

Our study had several limitations which should be taken into consideration. This is a retrospective non-randomized cohort study and some parameters were not strictly regulated, thus is susceptible to observer and selection bias. For example, ECG-controlled tube current modulation was not adapted in the RGH group and led to more radiation dose and more evaluable cardiac phases as compared to the PGA group. The current PGA implementation itself has inherent limitations because of the lack of multi-sector reconstruction capability. Also, a mid-diastolic trigger centered at 75% of the R-R interval is used to align the imaging window with diastole for patients with HR ≤75 bpm for PGA. At higher HR (>75 bpm), an end-systolic imaging window centered at 40% of the R-R interval can be used to image the heart in a rest period composed of the reduced ejection, protodiastole ejection and isovolumetric relaxation. Thus, we did not investigate the robustness of the PGA technique for patients with HR >75 bpm. Besides, owing to diffuse, calcified, and atherosclerotic disease, the image quality of native coronary artery was not assessed although it was also clinically important in those patients. Additionally, we only evaluated image quality and not diagnostic accuracy, *i.e.*, for the detection of coronary artery bypass graft stenoses. It is not known whether sensitivity and specificity for stenosis detection would differ for scanning protocols using PGA and RGH technique. Although coronary CTA imaging of patients with prior coronary bypass surgery yields very accurate information about the state of the bypass grafts with a sensitivity of 97% and a specificity of 100% as compared to conventional coronary angiography for following CABG [3], [4], there are currently no catheter angiography data available for validation of the results of this study. Moreover, radiation exposure was estimated and not measured. Lastly, although we found better image quality in the PGA group, it cannot be ruled out that this result would become less significant if a larger sample size had been used.

In conclusion, with the 256-slice scanner for following bypass surgery, over 90% of segments received the good image quality score for both PGA and RGH techniques, and the overall image quality scores for RGH and PGA groups were 1.73±0.62 and 1.51±0.53, respectively. There was no significant difference in image noise, SNR and CNR between the two groups. The PGA group had better subjective image quality of all graft segments, arterial and venous bypass grafts and there was a significant difference in the image quality of middle body and distal anastomosis of SV grafts, proximal body and middle body of LIMA grafts in segment-to-segment comparison. While providing better image quality than RGH for patients with prescan HR >70 bpm, PGA for patients with prescan HR <70 bpm was associated with a 65.0% reduction in effective radiation dose. PGA technique should be recommended for CABG patients with HR <68 bpm and low HRV to lower patient radiation dose and provide better image quality. Further study to confirm the diagnostic accuracy of 256-slice PGA/RGH-CTCA as compared to conventional coronary angiography in patients with prior coronary bypass surgery is warranted.
