# Correction: Prospectively versus Retrospectively ECG-Gated 256-Slice CT Angiography to Assess Coronary Artery Bypass Grafts — Comparison of Image Quality and Radiation Dose

**DOI:** 10.1371/annotation/2d9c062e-1454-435d-b74a-4ce2cdaf5651

**Published:** 2013-06-04

**Authors:** Yi-Wei Lee, Ching-Ching Yang, Greta S. P. Mok, Wei-Yip Law, Cheng-Tau Su, Tung-Hsin Wu

There were typographical errors in Tables 1 and 4. Please see the following links to the corrected versions of Tables 1 and 4:

**Figure:**
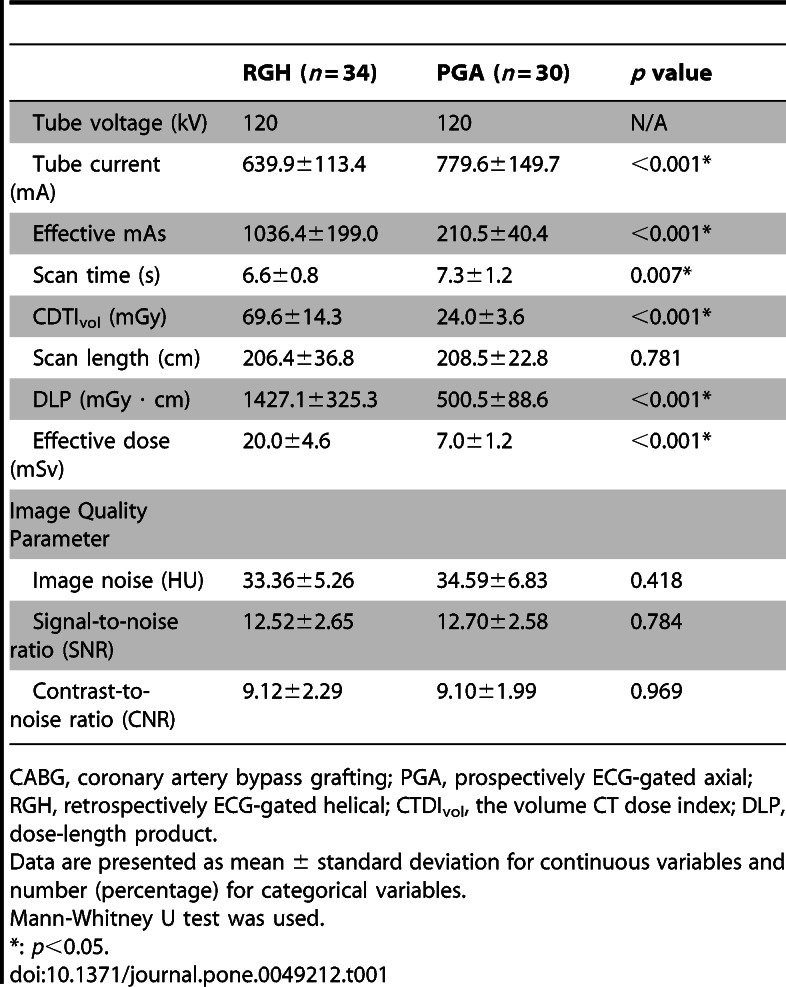


**Figure:**
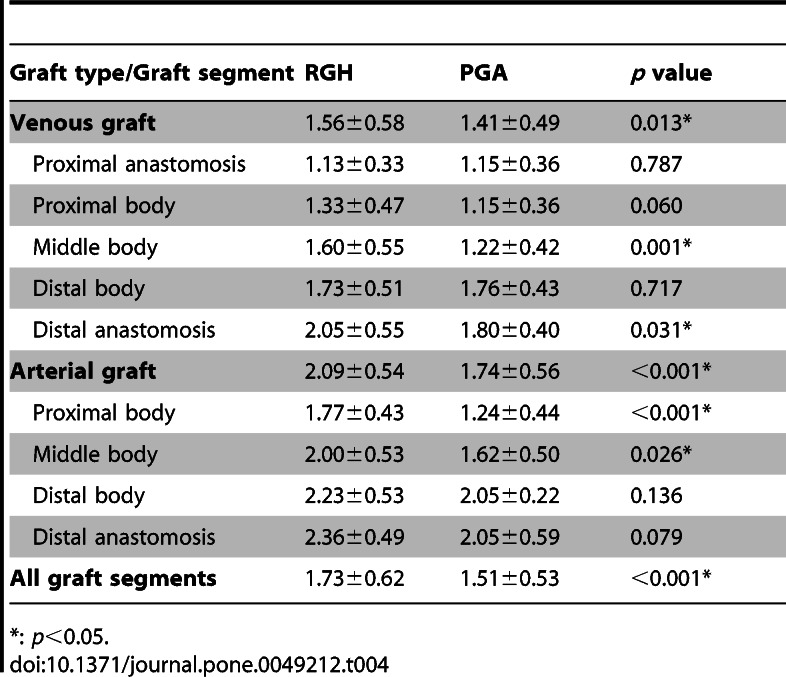


In the section titled, "CT Data Post-processing and Analysis", the first equation contains a word that is misspelled. The word "Imagenoise" should read "Image noise".

